# Chemical Composition and Biological Properties of *Rhododendron anthopogon* Essential Oil 

**DOI:** 10.3390/molecules15042326

**Published:** 2010-03-31

**Authors:** Gabbriella Innocenti, Stefano Dall’Acqua, Giuditta Scialino, Elena Banfi, Silvio Sosa, Khilendra Gurung, Mariagnese Barbera, Maria Carrara

**Affiliations:** 1 Department of Pharmaceutical Sciences, University of Padova, Via F. Marzolo, 5, 35131 Padova, Italy; E-Mail: stefano.dallacqua@unipd.it (S.D.); 2 Department of Life Sciences, Microbiology Section, University of Trieste, Via Fleming, 22, 34127 Trieste, Italy; E-Mails: scialino.giuditta@libero.it (G.S.); banfi@dvs.units.it (E.B.); 3 Department of Materials and Natural Resources, University of Trieste, Via A. Valerio 6, 34127 Trieste, Italy; E-Mail: silvio.sosa@econ.univ.trieste.it (S.S.); 4 Research and Development Center, Kathmandu, Nepal; E-Mail: khilendragurung@hotmail.com (K.G.); 5 Department of Pharmacology and Anaesthesiology, University of Padova, L.go E. Meneghetti 2, 35131 Padova, Italy; E-Mails: mb584@hutchison-mrc.cam.ac.uk (M.B.); maria.carrara@unipd.it (M.C.); 6 Hutchison-MRC Research Centre, Hills Road, Cambridge, CB2 0XZ, UK

**Keywords:** *Rhododendron anthopogon*, GC-MS, essential oil, antimicrobial activity, topical anti-inflammatory activity, antiproliferative activity

## Abstract

The essential oil of *Rhododendron anthopogon* was investigated by GC-MS, and seventeen compounds (representing approximately 98% of the oil) were identified. The major components of the aerial parts of the oil were the monoterpenes α-pinene, β-pinene, limonene and the sesquiterpene δ-cadinene. Biological studies revealed a weak topical anti-inflammatory activity; a significant killing effect against some Gram-positive reference strains: *Staphylococcus aureus*, *Enterococcusfecalis, Bacillus subtilis* was measured; *Mycobacterium tuberculosis* reference strain and a clinical isolate of *Candida*, *C. pseudotropicalis* were killed by as low as 0.04% (v/v) essential oil. Moreover, the oil was able to reduce cancer cell growth independently of the cell line and the treatment protocols used.

## 1. Introduction

Essential oils are valuable natural products used as raw materials in many fields, such as perfumes, cosmetics, aromatherapy, spices and nutrition [1,2]. There is an increasing world-wide interest in screening plants to study the biological activities of their oils with particular focus on their chemical, pharmacological and therapeutic properties [3,4,5,6,7,8].

In continuing our research on medicinal plants from Nepal [8,9,10,11], we considered the essential oil of *Rhododendron anthopogon* D. Don. (Ericaceae), an evergreen shrub growing at an altitude of 3,000 – 4,000 m a.s.l., harvested in several regions of Nepal. This plant is a national symbol in Nepal and is widely used as incense for its aromatic properties. Furthermore, its leaves and fresh flowers are used as tea by Himalayan healers to promote digestive heat, stimulate appetite and relieve liver disorders. *R. anthopogon* is also used for sore throat, common cold and lung problems [12]. The essential oil, Anthopogon oil, as it is usually referred to in Nepal, is obtained by steam distillation of the aerial parts of *R. anthopogon.* Also known as Sunpati oil, this oil is a good natural source of sweet herbal, a faintly balsamic essence [12]. *R. anthopogon* essential oil can be used on the skin and hair. According to Himalayan aromatherapy, this oil stimulates the nervous system and has been used for treating sore muscles and gouty rheumatic conditions [12].

Up to date few studies have been published about the chemical characterization [13,14,15,16] and the biological properties of this plant [17,18]. In this paper, we describe the chemical composition and the evaluation of topical anti-inflammatory, anti-microbial and antifungal activities of the essential oil of *R. anthopogon*. Furthermore, we also evaluate the anti-proliferative effect of the oil against three different human tumor cell lines.

## 2. Results and Discussion

### 2.1. Chemical analysis

The essential oil was extracted by steam distillation from the fresh aerial parts (leaves and flowers) of *R. anthopogon* and its chemical composition was determined by GC-FID and GC-MS. Almost all components of the oil were identified and their percentages are listed in order of their elution on the DB-5 column (Table 1). A total of 17 compounds were identified representing 97.8 % of all the components found in the sample. These percentages were calculated using normalization of peak areas without application of the response correction factor. 

The oil was characterized by a high amount of monoterpene hydrocarbons (76.1%), mainly α-pinene (37.4%), followed by β-pinene (16.1%), limonene (13.3%), and the sesquiterpene, δ-cadinene (9.1%). The composition of *R. anthopogon* essential oil was quite different from those of other *Rhododendron* species: the main components of *R. nivale* essential oil are δ-cadinene and α-cadinol [19], whereas β-pinene, camphene and δ-3-carene are the major constituents of the oil of *R. mucronulatum* [20]*.* The composition of *R. anthopogonoides* essential oil is also totally different; in fact, its main components are benzylacetone and selina-3,7-diene [21]. Furthermore, studies on essential oils from leaves of *R*. *dauricum* and *R. aureum* found that the dominant compounds were *trans*-caryophyllene and calarene, respectively [22].

**Table 1:** Chemical composition of *R. anthopogon* essential oil.

Compound	RI ^a^	RI ^b^	Identification method	%
α -Thujene	929	1018	1, 2	0.21 ± 0.01
α -Pinene	937	1032	1, 2, 3	37.40 ± 0.16
Camphene	954	1076	1, 2, 3	0.23 ± 0.02
β-Pinene	979	1118	1, 2, 3	15.98 ± 0.11
β-Myrcene	984	1174	1, 2, 3	1.10 ± 0.04
*p*-Cymene	1023	1280	1, 2, 3	2.60 ± 0.06
Limonene	1030	1203	1, 2, 3	13.3 ± 0.2
*cis*-Ocimene	1050	1262	1, 2	5.32 ± 0.24
γ-Terpinene	1062	1255	1, 2, 3	1.47 ± 0.07
α-Copaene	1364	1495	1, 2	0.74 ± 0.02
*trans*-β-Cariophyllene	1420	1612	1, 2, 3	2.26 ± 0.06
α-Humulene	1449	1687	1, 2, 3	0.20 ± 0.01
*Allo*-Aromandrene	1461	1661	1, 2	0.23 ± 0.01
Germacrene D	1480	1726	1, 2	1.77 ± 0.07
α-Amorphene	1485	1675	1, 2	3.15 ± 0.11
α-Muurolene	1499	1740	1, 2	2.74 ± 0.12
δ-Cadinene	1524	1773	1, 2	9.10 ± 0.15

^a^ Kovats retention index calculated on DB-5 column; ^b ^Kovats retention indices calculated on HP INNOwax column; 1: Kovats retention index, 2: mass spectrum, 3: co-injection with authentic compounds.

### 2.2. Anti-inflammatory activity

The topical anti-inflammatory activity of *R. anthopogon* essential oil was evaluated using the Croton oil ear test in mouse. The results, reported in Table 2, show that only the highest dose of oil (4,000 μg/cm^2^) significantly inhibited the oedema formation (40% of control). This activity was comparable with that induced by indometacin, chosen as a reference compound, but at a dose 40 times higher.

**Table 2:** The topical anti-inflammatory activity of *R. anthopogon* essential oil and indomethacin on croton oil induced ear oedema in mice.

Group	Dose (µg/cm^2^)	Animal number	Weight mg ± E.S.	Oedema inhibition %
Controls	--	10	7.0 ± 0.3	--
*R. anthopogon* oil	1,000	10	6.0 ± 0.6	14
*R. anthopogon* oil	4,000	10	4.2 ± 0.2*	40
Indomethacin	100	10	3.0 ± 0.3*	57

* Significantly different from control (p < 0.05).

### 2.3. Anti-microbial activity

The anti-microbial activity of *R. anthopogon* essential oil was evaluated by reference micro-dilution assays against a series of Gram positive and Gram negative reference strains, against *Mycobacterium tuberculosis* reference strain and against a series of 15 strains, all clinical isolates, of *Candida* spp. Table 3 reports the MICs of *R. anthopogon* essential oil against bacteria, MIC range of reference drugs is also reported. An interesting killing activity was measured against *S. aureus* (2.5% v/v), *E. fecalis* (1.25% v/v), *B. subtilis* (0.04% v/v) and *M. tuberculosis* (0.04% v/v). It is well known that the anti-microbial activity of essential oils is strictly connected to their chemical composition [23]. Therefore, the detected anti-microbial properties of this essential oil could be due to the relatively high concentration of α-pinene (37.4%), β-pinene (16%) and limonene (13.3%), which are believed to actively inhibit the growth of microorganisms [24].

Table 4 reports the MICs measured by a reference microdilution assay after 24 and 48 h incubation time, against fungi, all clinical isolates, some of them resulting drug-resistant strains. Most clinical strains of *Candida* spp were shown to be susceptible to the *R. anthopogon* essential oil at doses comparable with reference antifungal drugs, in particular a clinical isolate of *C. pseudotropicalis* with a MIC of 0.04% v/v after 48 h incubation.

**Table 3:** Antibacterial activity of *R. anthopogon* essential oil, evaluated by microdilution assay.

Strains	MIC (% v/v)
*S. aureus ATCC 25923*	2.5
*E. fecalis* ATCC 29216	1.25
*B. subtilis* ATCC 6633	0.04
*E. coli* ATCC 25922	>5
*P. aeruginosa* ATCC 27753	>5
*M. tuberculosis* H37 Rv	0.04

Ampicillin, ciprofloxacin, isoniazide, rifampicin MIC ranges: 0.05–0.1 g/mL

**Table 4:** Antifungal activity of *R. anthopogon* essential oil, evaluated by microdilution assay.

Clinical Strains	24 h MIC (% v/v)	48 h MIC (% v/v)
*Candida albicans*	0.08-0.04	0.3-0.15
*C. albicans*	0.15	0.3
*C. albicans*	0.15	0.6
*C. albicans*	0.6-0.3	0.6
*C. albicans*	0.3	2.5
*C. albicans*	0.3	1.25
*Candida glabrata*	0.04	0.15
*C. glabrata*	0.15	0.6
*C. glabrata*	1.25	5
*C. glabrata*	2.5	>5
*Candida tropicalis*	1.25-0.6	1.25
*C. tropicalis*	0.3	1.25
*Candida parapsilosis*	0.3	1.25
*C. parapsilosis*	0.3	0.6
*C. pseudotropicalis*	0.04	0.04

Miconazole MIC range: 0.125–64 g/mL; Amphotericin B MIC range: 0.25–4 g/mL.

### 2.4. Anti-proliferative activity

Evaluation of the anti-proliferative activity of *R. anthopogon* essential oil was performed on three human adenocarcinoma cell lines: ovarian (2008), cervix (A-431) and colon (LoVo) using two different treatment protocols.

a) The first treatment protocol (3 + 21 or 24 h treatment) was used both to identify a range of doses for the time-course experiments and to detect possible raw effects. Generally, when the cells were exposed for 3 h at 200, 400 and 600 μg/mL of *R. anthopogon* essential oil and then incubated with culture medium for 21 h, the results revealed a dose-dependent cytotoxic effect. 

**Figure 1:**
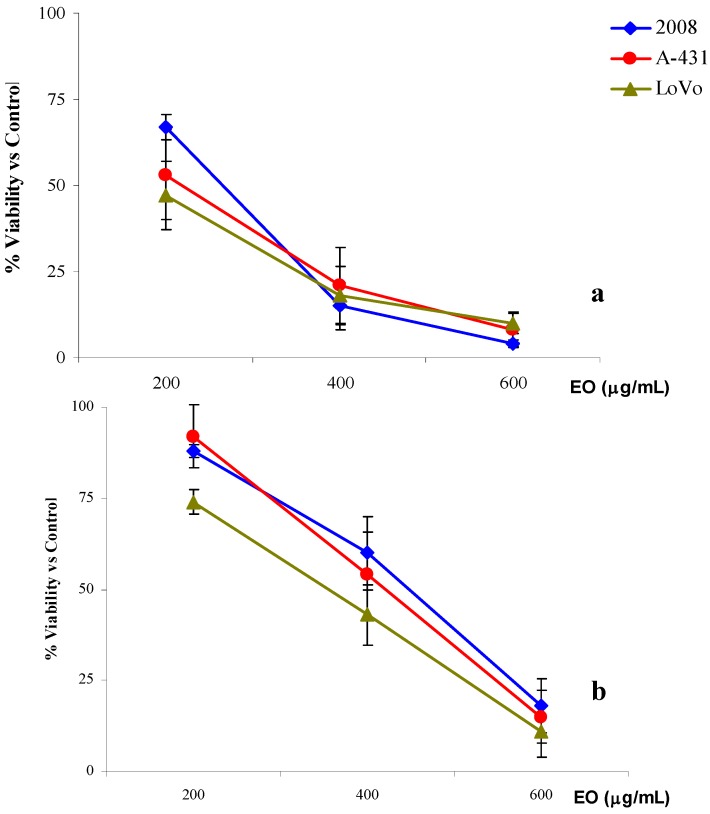
% cell viability *versus* control of 2008, LoVo and A-31 cells treated with *R. anthopogon* essential oil (EO) for 3h + 21h incubation in culture medium (a) or 24h (b).

In particular, for all the three cell lines, a very significant cell growth inhibition was obtained with the two highest concentrations used. As shown in Figure 1a, very similar cell proliferation decreases were observed on 2008, A-431 and LoVo. Comparable IC_50 _values were also experimentally obtained for the three cell lines: 246.1 (223.4–271.2) g/mL for 2008, 213.5 (184.6–247.0) g/mL for A-431and 236.6 (202.2–276.8) μg/mL for LoVo (Table 5). When the exposure time was extended to 24 h using 100, 200 and 400 μg/mL of essential oil, results similar to those of previous assays were obtained (Figure 1b). The highest concentration of the essential oil induced a remarkable inhibition of cell growth and the values of IC_50 _were very similar to those previously obtained: 224.0 (196.9-254.9) µg/mL for 2008 cells, 218.6 (186.9–255.6) µg/mL for A-431cells and 217.6 (187.9–252.0) μg/mL for LoVo (Table 5).

**Table 5:** IC_50_ values and L.C. calculated by exposing 2008, LoVo and A-431cells to *R. anthopogon* essential oil with different treatment protocols.

	Time Treatment				
	3 h + 21 h	24 h		48 h	72 h
	Concentrations μg/mL				
	200, 400, 600	100, 200, 400	25, 50, 100, 200		
**2008**	246.1	224.0	186.4	154.2	159.7
	(223.4-271.2)	(196.9-254.9)	(158.4-219.5)	(131.8-180.3)	(139.8-182.5)
**LoVo**	213.5	218.6	146.2	108.5	118.1
	(184.6-247.0)	(186.9-255.6)	(128.3-166.6)	(64.6-182.3)	(60.6-230.1)
**A-431**	236.6	217.6	75.3	41.5	41.3
	(202.2-276.8)	(187.9-252.0)	(37.9-149.3)	(21.0-81.9)	(25.6-66.5)

These first two treatments did not show any difference in the results regardless of the type of cell lines used and exposure time. In fact, all IC_50 _values were at least comparable if not almost identical.

**Figure 2:**
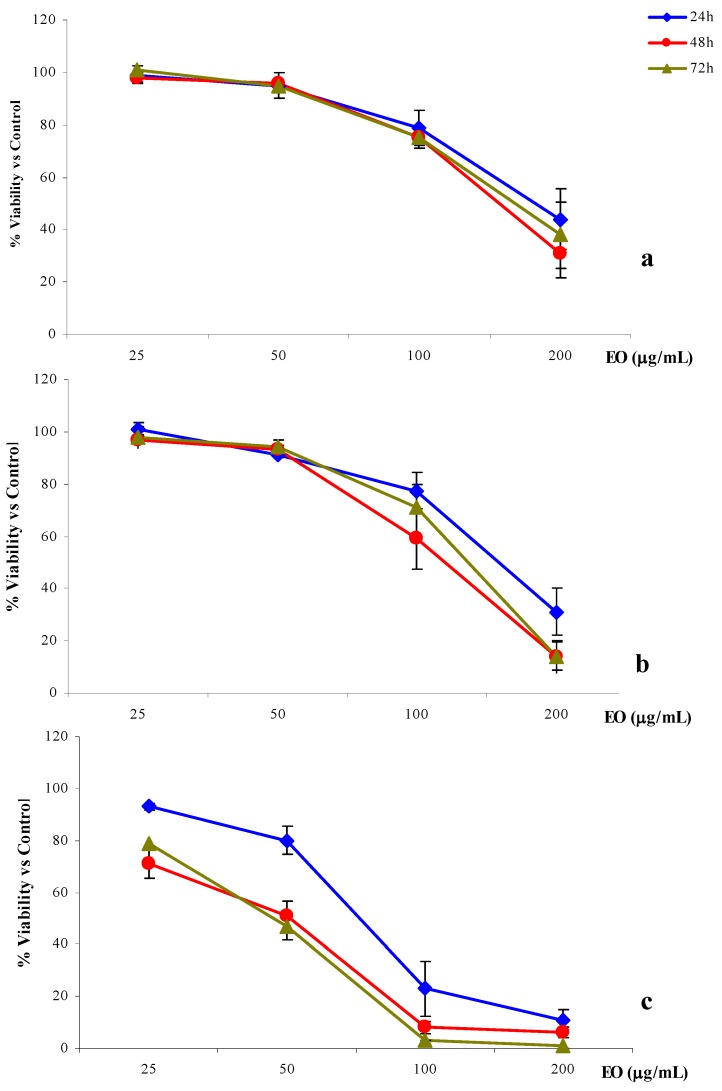
% cell viability *versus* control of 2008 (a), LoVo (b) and A-431(c) cells treated with *R. anthopogon* essential oil (EO) for 24, 48 or 72h.

b) The second treatment protocol (24, 48, 72 h treatment) was used to clarify the role played by the time of exposure. In this case the cells were seeded at a low density and treated for 24, 48 or 72 h and the increase of the cytotoxic effects of the essential oil occurred in a dose-dependent manner. However, the results showed completely different trends, depending on the exposure time and, particularly, on the cell line treated (Figure 2).

Among the three cell lines considered, 2008 was the most resistant to the anti-proliferative activity of the essential oil. In fact, the three IC_50 _values calculated for the different exposure times [186.4 (158.4–219.5) μg/mL, 154.2 (131.8–180.3) and 159.7 (139.8–185.2) μg/mL for 24, 48 and 72 h, respectively], were remarkably higher than those obtained with the two other cell lines (Table 5). Furthermore, a significant inhibition of cell proliferation was obtained only by exposing the cells to the highest concentration (200 μg/mL) without any evident correlation with the exposure time (Figure 2a).

Compared to 2008 cells, LoVo cells were slightly more sensitive to the anti-proliferative activity of the *R. anthopogon* essential oil, even though a significant inhibition of cell viability was reached only with the highest concentration (200 μg/mL). In the case of the 48 h treatment, the IC_50_ value calculated for the colon cells was 30% lower than that obtained with the ovarian cells [108.5 (64.6–182.3) μg/mL *versus* 154.2 (131.1–180.3) μg/mL] (Table 5). In this case the exposure time seems to play an important role. In fact, while a moderate inhibition of cell viability was obtained after 24 h treatment, prolonged treatments (48 h or 72 h) resulted in significant inhibition of viability (Figure 2.b).

The cervix adenocarcinoma cell line, A-431, was significantly more sensitive to the essential oil in comparison to either LoVo or, especially, the 2008 cell line. In particular, the essential oil was able to induce a significant inhibition of viability already at 100 μg/mL (Figure 2.c). After 24 h of exposure to the oil, the IC_50_ obtained for A-431 was more than two-fold lower than the one observed in the case of 2008 cells, in the same conditions [75.3 (37.9–149.3) μg/mL *versus* 186.4 (158.4–219.5) μg/mL]. Comparing the 48 h and 72 h treatments on 2008 and A-431 cells performed under the same conditions, the IC_50_ calculated on A-431 was found to be more than three times lower [41.5 (21.0–81.9) μg/mL *versus* 154.2 (131.1–180.3) μg/mL and 41.3 (25.6–66.5) μg/mL *versus* 159.7 (139.8–185.2) μg/mL; Table 5]. On this cell line the anti-proliferative activity of the *R. anthopogon* essential oil depended on the time of exposure (Figure 2c). The cell proliferation following 24 h treatment was remarkably different than that obtained with the 48 h and 72 h treatments, which showed, in both cases, a decrease in IC_50_ of 45% [41.5 (21.0–81.9) and 41.3 (25.6–66.5) μg/mL after 48 h and 72 h, respectively *versus* 75.3 (37.9–149.3) μg/mL after 24 h; Table 5].

## 3. Experimental

### 3.1. Plant material

Aerial parts including leaves and flowers of *R. anthopogon* were collected from the Rolwaling area of Dolakha district, Nepal (4,000–4,500 m a.s.l.), during August-September 2005. The plant was identified by one of the authors (K. G.) and a voucher specimen was deposited at Herbs Production and Processing Co Ltd., (HPPCL), Kathmandu, as n° RA0805.

### 3.2. Isolation of essential oil

The essential oil was obtained from fresh aerial parts by hydrodistillation with a Clevenger-type apparatus in the laboratories of HPPCL. The oil was stored at 4 °C in a sealed brown vial until analysis.

### 3.3. GC-FID and GC-MS analysis

The oil was analysed by GC on an Agilent 6840N gas chromatograph equipped with a FID detector. The separation was achieved using a fused-silica-capillary column, DB-5, (30 m × 0.25 mm i.d. × 0.25 μm film thickness, from J&W Scientific, Folsom, CA, USA). The analysis was carried out in the following conditions: injector and detector temperatures, 230 °C and 290 °C, respectively; carrier gas, He at a flow rate of 0.8 mL/min; split ratio 1:10; injection volume 1 μL. Temperature programme: 60 °C with 3 min initial hold, and then to 280 °C at a rate of 3 °C/min, and finally held isothermally for 5 min. Analysis was also run by using a fused silica HP INNOwax polyethylenglycol capillary column (30 m × 0.25 mm i.d. × 0.25 μm film thickness); the temperature programme for this column was from 60 °C to 260 °C .

GC-MS analyses were performed on a HP6890 gas chromatograph equipped with a HP 5973 mass selective detector (MS), equipped with a fused-silica-capillary column, DB-5, (30 m × 0.25 mm i.d. × 0.25 μm film thickness). Ionization mode was electronic impact at 70 eV. Mass range was set from 40 to 450 Da. Gas chromatographic conditions were the same as described for GC-FID.

Components were identified by comparison of their mass spectra with those of Wiley Library and confirmed by comparing the retention indexes (relative to C_6_-C_24_ *n*-alkanes) with authentic standards from Sigma-Aldrich (Milano, Italy) or values from the literature. The percentage composition of the oil was calculated from the GC peak areas using the normalization method without correction factors. The data are reported as mean value of four oil injections 

### 3.4. Assay for topical anti-inflammatory activity

*Animals*** –**Male CD-1 mice (28–32 g), obtained by Harlan Italy (Udine, Italy), were maintained under standard conditions (light/dark cycle of 12 h; humidity: 55–60%; room temperature: 22 ± 2 °C) with food and water *ad libitum*. The study was carried out in accordance with the Guide for the Care and Use of Laboratory Animals as adopted and promulgated by U.S. National Institute of Health.

*Croton oil-induced mouse ear oedema*** – **Oedema was induced as previously described by Tubaro *et al*. [25]. Cutaneous inflammation was induced by application of 15 µL of an acetone solution containing the irritant (80 µg of croton oil, Sigma Aldrich; Milan, Italy) to the inner surface of the right ear of the anaesthetised mice (145 mg/kg ketamine hydrochloride, i.p., obtained by Virbac S.r.l., Milano, Italy). *R. anthopogon* essential oil was dissolved in the irritant solution and applied topically. The left ear received the vehicle. As reference, indomethacin (100 μg/cm^2^; Sigma Aldrich, Milan, Italy) was used. After six hours, the mice were sacrificed and a plug (6 mm Ø) was removed from both the ears. Inflammation was measured as oedema formation and quantified by the weight difference between the treated and the untreated (opposite) ear samples. The anti-inflammatory activity was expressed as percent inhibition of oedema in mice treated with *R. anthopogon* essential oil and indomethacin in comparison with control mice treated with the irritant alone. The results are reported as mean values ± ES for six animal per group. Differences between control and treatment groups were tested for significance p < 0.05 using Student’s *t*-test.

### 3.5. Anti-microbiological susceptibility testing

Anti-microbial activity was determined in a preliminary evaluation by an agar disc-diffusion Kirby Bauer test, absorbing different amounts of *R. anthopogon* essential oil onto sterile, 6 mm diameter paper disks and measuring the inhibition zone diameter obtained after overnight incubation in agar medium [26]. Gram positive and Gram negative bacteria: *Staphylococcus aureus* (ATCC 20202), *Enterococcus fecalis* (ATCC 29216), *Bacillus subtilis* (ATCC 6633), *Escherichia coli* (ATCC 25922), *Pseudomonas aeruginosa* (ATCC 27753) were evaluated to measure the anti-bacterial activity; a total of six strains of *Candida albicans* and nine strains of *Candida* spp, all clinical isolates, were selected to test the antifungal activity. Furthermore, *Mycobacterium tuberculosis* reference strain H37Rv was employed to determine the anti-tubercular activity.

The minimal inhibitory concentration (MIC) values were measured for the bacterial strains using standard broth microdilution assay in Mueller Hinton broth [27]. Each test was carried out twice in duplicate. Ampicillin and ciprofloxacin were chosen as positive control to determine the sensitivity of each bacterial strain. 

*In vitro* anti-tubercular activity was evaluated by measuring *R. anthopogon* essential oil MIC twice in duplicate experiments by MRA, a reliable one-week duration micro-dilution Resazurin assay, as previously described [28,29]; Isoniazide and Rifampicin were used as reference drug controls. 

*In vitro* antifungal activity was assessed against 15 *Candida* spp. clinical isolates using a microdilution RPMI reference method [30,31]. Miconazole and amphotericin B were used as reference test compounds. Each MIC was determined twice in duplicate after an incubation time of 24 and 48 h.

### 3.6. Anti-proliferative activity on cancer cells

*R. anthopogon* essential oil was dissolved in DMSO and diluted in culture medium to obtain a stock solution, which was stored at –20 °C. The final stock solution composition was: 1% DMSO, 9% essential oil and 90% culture medium. Each assay was performed on three human adenocarcinoma cell lines: ovarian (2008), cervix (A-431) and colon (LoVo). 2008 and A-431 were maintained in RPMI 1640 medium, LoVo in HAM’s F-12 medium; both culture media were supplemented with 10% heat-inactivated FCS, 1% antibiotics (both from Biochrom KG Seromed) and 1% 200 mM glutamine (Merck**)**.

Anti-proliferative activity was evaluated by two different treatment protocols:

a) In the first, cells were seeded in 96-wells tissue plates (2008 and A-431 8 × 10^3^ cells/well and LoVo 10^4^ cells/well) (Falcon). Following overnight incubation at 37 °C and 5% CO_2_, the cells were exposed for 3 or 24 h at concentrations of *R. anthopogon* essential oil ranging from 100 to 600 μg/mL. After exposure, the cells treated for 3h were washed and incubated in culture medium for 21 h.
b) In the second, cells were seeded in 96-well tissue plates (2008 and A-431, 5 × 10^3^ cells/mL, LoVo 8 × 10^3^ cells/mL) (Falcon). After 24 h, the cells were exposed to four concentrations of essential oil (25, 50, 100 and 200 μg/mL) for either 24, 48 or 72 h.

Cell proliferation was assessed by the MTT salts reduction assay: MTT salts in PBS (20 μL of 5 mg/mL solution) were added to each well 4 h before the end of the treatment and the plates were incubated at 37 °C. At the end of the treatment, the culture media was discarded and the dark blue crystals were dissolved in DMSO (150 μL/well). Absorbance was measured on a micro-culture plate reader (Titertek Multiscan) using 570 nm and 630 nm as test and reference wavelengths, respectively. For each assay, at least five experiments were performed in triplicate. Statistical analysis was carried out using Student's *t*-test. IC_50_ and 95% confidence limits were calculated using GraphPad 3.0 software.

## 4. Conclusions

To the best of our knowledge, this is the first report on the chemical composition and biological activities of *R*. *anthopogon* essential oil. The results obtained indicate that, although topical anti-inflammatory activity was obtained only at very high concentrations, a remarkable anti-microbial activity was detected against *B. subtilis* and *M. tuberculosis.* Moreover, the *R. anthopogon* essential oil inhibited most clinical strains of *Candida* spp at doses comparable with reference antifungal drugs and the strongest activity was found against a clinical isolate of *C. pseudotropicalis*. It is well known that the anti-microbial activity of essential oils is strictly connected to their chemical composition [23]. Thus, the detected ant-imicrobial properties of this essential oil could be due to the most abundant components α-pinene, β-pinene and limonene, which are believed to be active in growth inhibition of microorganisms [24]. 

The interesting anti-microbial effect registered may support at least in part the traditional use in Himalayan folk medicine of *R. anthopogon* essential oil*.* In addition, the anti-proliferative activity of *R. anthopogon* essential oil was also observed against tumor cell lines. For treatments up to 24 h, the essential oil did not exert very significant anti-proliferative effects and no differences were found among the three cell lines or the treatment protocols. When the exposure time was extended to 48 h or more, the essential oil exerted a remarkable anti-proliferative effect on A-431 cells, which appeared to be much more sensitive than the other two cell lines. In fact, 100 μg/mL essential oil were enough to significantly inhibit the growth of A-431 cells, whereas the concentration had to be doubled to observe similar effects on 2008 and LoVo cells. Considering the chemical composition and anti-proliferative activity of essential oils, it has been proposed that the activity is not always related to their major constituents [32]. In this case, the most abundant components (α- and β-pinene) are not considered highly cytotoxic [33]; however, limonene is known for its effects against tumor cell line [34,35].

Since the essential oil is a complex natural mixture of volatile secondary metabolites, its biological activity may be related to synergistic interaction of both the major and minor components within the oil. Unravelling the chemical composition and biological activities of the aerial parts of *R. anthopogon* essential oil could contribute to a better utilization of this raw material. Essential oils are quite simple to obtain from fresh plants and could become an economical resource for many countries with high biodiversity, such as Nepal.
